# Current Models for Transcriptional Regulation of Secondary Cell Wall Biosynthesis in Grasses

**DOI:** 10.3389/fpls.2018.00399

**Published:** 2018-04-04

**Authors:** Xiaolan Rao, Richard A. Dixon

**Affiliations:** ^1^BioDiscovery Institute and Department of Biological Sciences, University of North Texas, Denton, TX, United States; ^2^BioEnergy Science Center, United States Department of Energy, Oak Ridge, TN, United States; ^3^Center for Bioenergy Innovation, United States Department of Energy, Oak Ridge, TN, United States

**Keywords:** secondary cell wall, secondary cell wall regulation, transcription factor, grasses, lignin biosynthesis

## Abstract

Secondary cell walls mediate many crucial biological processes in plants including mechanical support, water and nutrient transport and stress management. They also provide an abundant resource of renewable feed, fiber, and fuel. The grass family contains the most important food, forage, and biofuel crops. Understanding the regulatory mechanism of secondary wall formation in grasses is necessary for exploiting these plants for agriculture and industry. Previous research has established a detailed model of the secondary wall regulatory network in the dicot model species *Arabidopsis thaliana*. Grasses, branching off from the dicot ancestor 140–150 million years ago, display distinct cell wall morphology and composition, suggesting potential for a different secondary wall regulation program from that established for dicots. Recently, combined application of molecular, genetic and bioinformatics approaches have revealed more transcription factors involved in secondary cell wall biosynthesis in grasses. Compared with the dicots, grasses exhibit a relatively conserved but nevertheless divergent transcriptional regulatory program to activate their secondary cell wall development and to coordinate secondary wall biosynthesis with other physiological processes.

## Introduction

The plant cell wall is a structural layer located outside of the cell membrane that provides the physical strength to maintain cell shape against gravity (Taiz and Zeiger, 1998). There are two types of cell wall, primary and secondary. The primary cell wall is a thin layer with considerable flexibility for extension, and is formed in most plant cells. In contrast, the secondary cell wall is a thicker layer deposited between the primary cell wall and the cell membrane, and is formed in specialized types of cells such as tracheid/vessel elements and fibers (Taiz and Zeiger, 1998; Cosgrove and Jarvis, 2012). Secondary cell walls play a pivotal role during plant development and are involved in resistance to abiotic/biotic stresses (Houston et al., 2016). At the same time, cell wall recalcitrance, resulting in large part from the complex cross-linked matrix of the lignified secondary cell wall, is the major barrier in conversion of biomass into biofuel (Himmel et al., 2007; Pauly and Keegstra, 2010). Plants in the grass (Poaceae) family supply the most abundant, renewable sources of both nutrition and sustainable energy. Therefore, knowledge of grass secondary wall regulation can be applied for genetic modification to improve the quality of food, forages and fuel crops that sustainably supply economic and ecological benefits.

Transcriptional regulation in secondary wall formation has been extensively elucidated in the dicot model species *Arabidopsis thaliana* (Zhong and Ye, 2015). However, details of the regulatory network in grass secondary wall formation are still under investigation. The emergence of secondary cell walls in plants occurred about 430 million years ago, as an adaptation for colonizing from ocean to dry land (Li and Chapple, 2010). Around 140–150 million years ago, monocots achieved divergence from the dicot ancestor (Chaw et al., 2004). Subsequently, particular classes of transcription factors (TFs) have expanded in monocot lineages, including the R2R3 MYB TF class to which many secondary wall-regulators belong (Rabinowicz et al., 1999; Zhao and Bartley, 2014). The evolutionary history suggests that grasses may share conservation of secondary cell wall regulation with dicots to some degree, but present their unique aspects. Recent evidence indirectly or directly supports this view. This review focuses on current advances in secondary cell wall regulation in grasses, and discusses the commonalities and the differences between grasses and dicots.

## Crosstalk Between Secondary Wall Synthesis and Other Physiological Processes

Establishment of secondary cell walls is not an independent event, but involves crosstalk with other biological processes. First, secondary wall accumulation is determined by sugar levels in the plant controlled by light and the circadian clock (Rogers et al., 2005). Plants have to maintain a balance between carbon supply captured through photosynthesis and carbon assimilation, which converts carbon resources into cell wall polymers (Smith and Stitt, 2007; Loque et al., 2015). Second, secondary walls are deposited in specialized cells that have ceased growth and achieved their final cell shape (Cosgrove and Jarvis, 2012). The events of cell-cycle exit and cell wall remodeling occur at the initial stage of secondary wall formation through differential regulation of cell cycle controllers and wall-modifying enzymes, respectively (Goulao et al., 2011; Didi et al., 2015; Polyn et al., 2015). In the process of tracheary element differentiation, secondary cell wall biosynthesis is required to be tightly coupled with programmed cell death (PCD) (Groover and Jones, 1999; Ohashi-Ito et al., 2010). Third, a strictly coordinated biosynthetic program is observed among individual secondary wall components including cellulose, xylan and lignin, which leads to the proper assembly of the secondary wall. Finally, the lignin biosynthesis pathway shares common intermediates with other secondary metabolism pathways such as flavonoid biosynthesis (Dixon et al., 2013), allowing plants to recruit controllers to shift the metabolic flow upon demand (Bhargava et al., 2010). To achieve this coordination, plants have employed a limited number of TF families to constitute a complex regulatory network that is capable of coordinating secondary cell wall biosynthesis with other physiological processes.

## Tfs Involved in Grass Secondary Wall Formation

### SWNs as Ancestral Master Switches for the Secondary Wall Program

A subgroup of NAC TFs, called secondary wall NACs (SWNs), function as top-level master switches for secondary cell wall biosynthesis. Diverse SWN orthologs exist in vascular plants, with first appearance in *S. moellendorffii* (Zhong et al., 2010a; Yao et al., 2012; Nakano et al., 2015). It has been considered that vascular plants may have employed these ancestral NACs via duplication for controlling secondary wall biosynthesis at the early stage of colonization of the land (Zhong et al., 2010a; Yao et al., 2012; Nakano et al., 2015).

Secondary wall NACs can be divided into four clades, according to their protein alignment (**Supplementary Figure S1**). Arabidopsis SWNs specifically expressed in vessels and fibers belong to clades I to III (called VNDs) and clade IV (called NST/SND), respectively (Zhong et al., 2010a). In Arabidopsis, AtVND6 and AtVND7 are responsible for determining tracheary element differentiation through controlling both secondary wall thickening and PCD in vessels (Ohashi-Ito et al., 2010; Yamaguchi et al., 2010), while AtNST1 and AtSND1 (also named as NST3) redundantly activate the whole secondary wall program in fiber cells (Zhong et al., 2006, 2007b; Mitsuda et al., 2007). The conserved function of SWN orthologs has been observed in many other dicots such as *Medicago* and poplar (Zhao et al., 2010; Zhong et al., 2010b; Wang et al., 2011), and in grasses including rice, maize, *Brachypodium*, and switchgrass (Zhong et al., 2011, 2015; Valdivia et al., 2013; Yoshida et al., 2013; Xiao et al., 2017). The exogenous overexpression of rice, maize and switchgrass SWNs in the Arabidopsis *nst1 snd1* double mutant can rescue the deficit of secondary wall development, and the endogenous overexpression of SWNs in rice, maize, and *Brachypodium* leads to secondary cell wall thickening and an upregulation of secondary wall-related genes (Zhong et al., 2011, 2015; Valdivia et al., 2013; Yoshida et al., 2013; Xiao et al., 2017). SWNs from rice, maize, *Brachypodium*, and switchgrass are capable of directly inducing the expression of secondary wall biosynthesis genes in Arabidopsis through binding to the SNBE (secondary wall NAC binding element) motif in the target gene’s promoters (Zhong et al., 2006, 2011, 2015; Valdivia et al., 2013). Moreover, an upregulation of PCD genes was observed following endogenous/exogenous overexpression of SWNs in clades I, II and III, but not of SWNs in clade IV, in both Arabidopsis and grasses (Zhong et al., 2011, 2015; Valdivia et al., 2013).

Though highly conserved functions of SWNs are shared in vascular plants, some differences in expression pattern and regulatory mechanisms of SWNs have been detected in grasses and dicots. Unlike the differentiation of spatial expression in Arabidopsis, SWNs in all four clades display a similar expression pattern in all the secondary wall-enriched cells including xylem vessels and cortical fibers in rice, maize, *Brachypodium*, and switchgrass (Zhong et al., 2011, 2015; Valdivia et al., 2013; Yoshida et al., 2013; Xiao et al., 2017). One explanation is that, in Arabidopsis, xylem fibers do not undergo cell death, as a result of the recruitment of SWNs in clade VI that activate secondary wall development but do not induce cell death, while the xylem vessel elements endure the coupled programs of secondary wall formation and cell death caused by SWNs in clades I, II, and III (Bollhoner et al., 2012). This may be not the case in grasses. Moreover, the regulation of SWNs displays different features among vascular plants. In the dicots Arabidopsis and *Medicago truncatula*, SND1 shows a relatively simple feedback- regulation that can be auto-activated via binding to its own promoter and negatively regulated by its downstream MYB TFs (Wang et al., 2011). In wood development in *Populus trichocarpa*, a more complex regulation is apparent. Full-size PtrSND1 members self-activate their own genes as that in Arabidopsis, whereas splice variants from PtrSND1-A2 and PtrVND6-C1 reciprocally cross-inhibit the expression of all SWN members in clades I to III and clade IV, respectively, without auto-repression of their cognate TFs (Li Q. et al., 2012; Lin et al., 2017). However, in rice, the alternatively spliced form of OsSWN2, which lacks the transcriptional activation domain, may participate in a negative feedback loop to OsSWN1 and its cognate gene OsSWN2 (Yoshida et al., 2013). Taken together, these observations suggest that, although grasses and dicots evolved from the last common ancestor to recruit SWNs as master switches in the secondary cell wall program, plants may utilize lineage-specific self-regulation of SWNs and different SWNs with functional specialization.

### MYB Clades as Activators in Secondary Wall Accumulation

Secondary wall NACs service as master switches in secondary wall biosynthesis though directly regulating the transcriptional changes in secondary wall-structural genes and downstream TFs. In Arabidopsis, AtMYB46 and its paralog AtMYB83 are specifically expressed in both fibers and vessels, and redundantly activate secondary cell wall enhancement (Zhong et al., 2007a; McCarthy et al., 2009). The MYB46/83 orthologs in rice, maize, and switchgrass are capable of rescuing the defect in secondary cell wall-thickening in the Arabidopsis *myb46/83* double mutant (Zhong et al., 2011, 2015). Similar to AtMYB46, constitutive overexpression of ZmMYB46, OsMYB46, and PvMYB46 in Arabidopsis led to ectopic secondary wall deposition in stem and increased the content of cellulose, xylan and lignin, without activating the PCD genes (Zhong et al., 2011, 2015; Ko et al., 2012; Kim et al., 2013). Moreover, AtMYB46 and its ortholog PvMYB46 share a high similarity in activation efficiency on eight *cis*-acting elements [named the secondary wall MYB-responsive element (SMRE)] to induce the expression of target genes involved in secondary wall-related cellulose, xylan, and lignin biosynthesis (Ko et al., 2012; Zhong and Ye, 2012; Kim et al., 2013; Zhong et al., 2015), indicating the conservation of MYB46 function in grasses and Arabidopsis.

Two clades of MYBs, MYB58/63, and MYB42/85 (**Supplementary Figure S2**), are considered to be lower-level regulators of secondary wall biosynthesis, whose promoters can be bound by MYB46/83. In Arabidopsis, AtMYB58/63 and AtMYB42/85 are grouped as lignin-specific regulators because they show exclusive activation of all lignin biosynthesis genes (except AtF5H) (Zhou et al., 2009; Zhao and Dixon, 2011). Consistently, overexpression of OsMYB58/63 or OsMYB42/85 in rice leads to an elevated lignin content in the vascular bundles and sclerenchyma (Hirano et al., 2013b), and overexpression of SbMYB60 (the ortholog of AtMYB58/63) activates the expression of lignin biosynthesis genes and increases the lignin concentration in the biomass (Scully et al., 2016). Both results indicate the positive roles of these grass TFs in lignin accumulation. However, OsMYB58/63 triggers the additional expression of secondary wall-related cellulose synthase genes (Noda et al., 2015), and SbMYB60 overexpression affects the abundance of cellulose and xylan in the cell wall (Scully et al., 2016), neither of which effects are associated with AtMYB58/63 in Arabidopsis (Zhou et al., 2009). Interestingly, promoter analysis reveals that OsMYB58/63 and its Arabidopsis ortholog AtMYB58/63 proteins display a similar capacity for recognizing their binding sites (called AC-elements) (Zhou et al., 2009; Noda et al., 2015). AtMYB58/63 can activate the expression of secondary wall-related cellulose synthase genes in rice, but not in Arabidopsis (Noda et al., 2015). One explanation is a change in promoter elements during evolution. AC elements are found in the promoter regions of lignin biosynthesis genes (except *F5H*) in Arabidopsis, but are absent in cellulose and xylan biosynthesis genes (Zhou et al., 2009; Zhao and Dixon, 2011). However, in rice, AC elements appear in the promoters of many secondary wall-related cellulose, xylan, and lignin biosynthesis genes (Zhou et al., 2009; Noda et al., 2015). Though rice and Arabidopsis MYB58/63 share commonalities of regulatory binding sites, the changed composition in *cis*-regulatory elements provides the basis for MYB58/63 to induce the biosynthesis program of all three secondary wall-components in rice but not in Arabidopsis.

Genes in the MYB55/61 and MYB103 clades (**Supplementary Figure S2**) are also positive regulators of secondary cell wall biosynthesis in Arabidopsis and grasses. The *atmyb61* mutant of Arabidopsis displayed fewer differentiated xylem vessels, and with reduced secondary wall-thickening, in the inflorescence stem (Newman et al., 2004; Romano et al., 2012). The target genes of AtMYB61 include a secondary wall-repressor *AtKNAT7*, a pectin methylesterase (*AtPME*) and *AtCCoAOMT7* (encoding the caffeoyl CoA 3-*O*-methyltransferase of monolignol biosynthesis) (Romano et al., 2012). In rice, the expression of OsMYB55/61 can be directly induced by OsSWN2 and OsSWN3 (Huang et al., 2015). OsMYB55/61 is capable of modulating lignin content in vascular bundles through activating lignin biosynthesis genes (at least *CAD2*) (Hirano et al., 2013b), and promoting secondary wall-related cellulose synthesis through binding to a GAMYB motif in the promoter region of *CESA* genes (Huang et al., 2015). OsMYB55/61 may contribute to the coordination of both cellulose and lignin biosynthesis in secondary wall formation.

**FIGURE 1:**
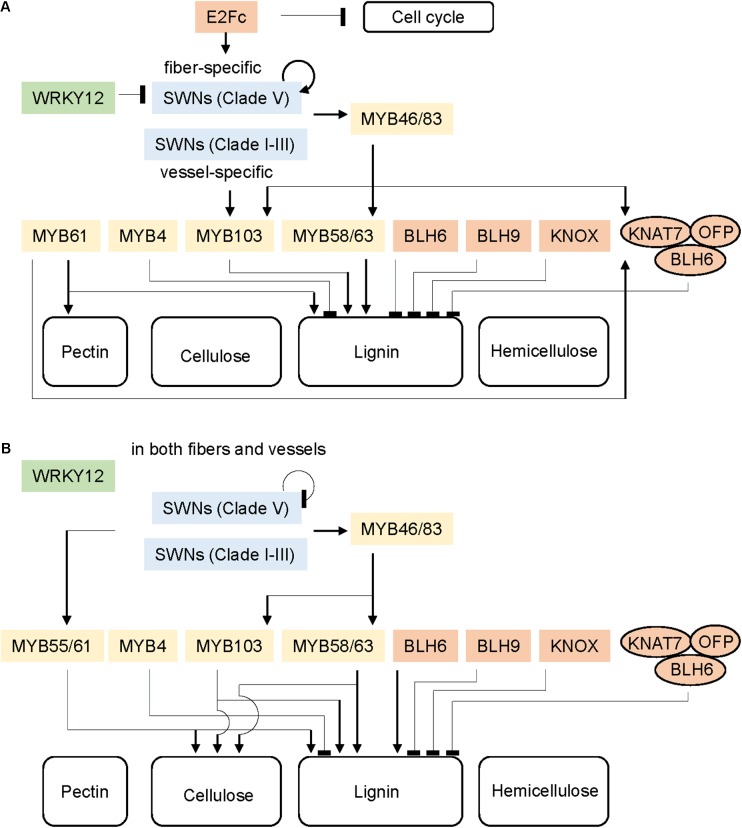
Schematic regulatory network of secondary cell wall formation in Arabidopsis **(A)** and grasses **(B)**. Colors represent transcription factor (TF) families: green, WRKY family; blue, SWN family; yellow, MYB family; red, other families. Arrows and bar at the ends of lines represent positive and negative transcriptional regulation, respectively. The ovals indicate a multi-protein complex.

AtMYB103, a direct transcriptional target of AtSWNs (SND1, NST1/2, and VND6/7) and AtMYB46/83, has been shown to interact with the promoter of a secondary wall-related cellulose synthesis gene *AtCESA8* in an Arabidopsis leaf protoplast transactivation system (Zhong et al., 2008; Ohashi-Ito et al., 2010; Yamaguchi et al., 2010). Interestingly, the Arabidopsis *atmyb103* mutant exhibits specific alteration of lignin composition via reduction of the expression of one lignin biosynthesis gene, *AtF5H*, but is not affected in the total lignin or cellulose content (Ohman et al., 2013). However, current evidence does not support the direct linkage of MYB103 and F5H in grasses. In rice, overexpression of OsMYB103 leads to increased cellulose content and enhanced secondary wall accumulation in sclerenchyma, whereas downregulating OsMYB103 results in decreased cellulose content and thinner cell walls in sclerenchyma, which together contribute to weakened mechanical strength of the culm (Hirano et al., 2013b; Yang et al., 2014; Ye et al., 2015). Microarray and transactivation experiments reveal that OsMYB103 significantly actives the expression of three secondary wall-related cellulose synthesis genes (*OsCESA4*, *OsCESA7*, and *OsCESA9*) and one secondary wall-related cellulose deposition gene (*OsBC1*) (Yang et al., 2014; Ye et al., 2015). Notably, a gibberellin (GA) signaling repressor (SLENDER RICE1, OsSLR1) shows physical interaction with OsMYB103 (Yang et al., 2014; Ye et al., 2015), and OsSWN2 and OsSWN3, which subsequently activate the expression of OsMYB55/61 (Huang et al., 2015). This suggests that OsMYB55/61 and OsMYB103 may play a role in controlling GA-mediated secondary wall biosynthesis (Yang et al., 2014; Huang et al., 2015; Ye et al., 2015) (**Figure 1**).

### A MYB Clade of Repressors of Secondary Wall Accumulation

Genes in the clade of MYB4/32 are proposed to be negative regulators of secondary wall biosynthesis in vascular plants (Zhao and Bartley, 2014) (**Supplementary Figure S2**). More accurately, the MYB4/32 clade should be considered as a controller that shifts the flux from the phenylpropanoid pathway to other metabolic pathways. In Arabidopsis, AtMYB4 suppresses the expression level of cinnamate-4-hydroxylase (*C4H*) and 4-coumarate:CoA ligase (*4CL*) genes, rather than other lignin biosynthesis genes, to control the accumulation of sinapate esters in response to ultraviolet-B (UV-B) irradiation (Jin et al., 2000). The AtMYB4 overexpressing Arabidopsis line has a decreased content of sinapate esters, with no effect on flavonoid composition (Jin et al., 2000). However, AtMYB7 and AtMYB32, two paralogs of AtMYB4, repress and induce genes involved in the flavonoid pathway, respectively (Preston et al., 2004; Fornalé et al., 2014); loss of function of AtMYB7 and AtMYB32 lead to notable induction of flavonoid content and alteration of pollen wall composition, respectively (Preston et al., 2004; Fornalé et al., 2014).

In contrast, AtMYB4 homologs in grasses have been observed to function in a more lineage-specific fashion for the regulation of lignin biosynthesis genes (Agarwal et al., 2016). In maize, ChIP-seq and coimmunoprecipitation (co-IP) assays revealed that ZmMYB11, ZmMYB31, and ZmMYB42 down-regulated different lignin biosynthesis genes, with the commonalities of *COMT* and *4CL2* (Vélez-Bermúdez et al., 2015). Exogenous overexpression of ZmMYB31 and ZmMYB42 in Arabidopsis redirected the phenylpropanoid flux by downregulating different lignin biosynthesis genes compared to maize (Fornalé et al., 2006, 2010; Sonbol et al., 2009; Vélez-Bermúdez et al., 2015). For example, ZmMYB31 and ZmMYB42 do not repress *ZmF5H* in maize, but do repress *AtF5H* in Arabidopsis, which causes decreased lignin S/G ratio (S, syringl units; G, guaiacyl units) (Sonbol et al., 2009; Fornalé et al., 2010; Vélez-Bermúdez et al., 2015). Comparatively, overexpression of PvMYB4 in tobacco results in significantly reduced expression of 10 lignin biosynthesis genes leading to reduced lignin content and higher S/G ratio, whereas overexpressing PvMYB4 in switchgrass does not alter lignin composition (Shen et al., 2012). This suggests that grasses may utilize different regulatory mechanisms using MYB4/32 clade TFs to balance the flux between the lignin and flavonoid pathways.

Interestingly, although MYB4/32 homologs in grasses predominantly recognize a conserved domain in the promoter of target genes, they inhibit different phenylpropanoid genes within grass lineages (Shen et al., 2012; Vélez-Bermúdez et al., 2015; Agarwal et al., 2016). In maize, sorghum and rice leaves, MYB4/32 syntelogs share the common target of *O*-methyltransferase (COMT1), but display divergent binding to the promoters of 4-coumarate-CoA ligase (4CL2), ferulate-5-hydroxylase (F5H), and caffeoyl shikimate esterase (CSE) (Agarwal et al., 2016). This suggests that genes in the MYB4/32 clade may have undergone sub-functionalization for fine-tuning of phenylpropanoid flux in some grass lineages (Agarwal et al., 2016).

### WRKY12 as Repressor

WRKY12, a member of group IIc of the WRKY TF family (Rushton et al., 2010; Phukan et al., 2016), has been shown to control pith cell maintenance through repressing lignification in pith cell walls in dicots (Wang et al., 2010; Gallego-Giraldo et al., 2016; Yang et al., 2016). In Arabidopsis, *M. truncatula*, *Populus*, and alfalfa (*M. sativa*) a reduction of WRKY12 expression leads to an enhanced and/or ectopic deposition of secondary cell walls in the pith cells of the stem (Wang et al., 2010; Gallego-Giraldo et al., 2016; Yang et al., 2016). Similarly, alteration in secondary cell wall deposition is observed on down-regulation WRKY12 orthologs in switchgrass and maize (Gallego-Giraldo et al., 2016). This suggests a conserved function of WRKR12 as a repressor of secondary cell wall accumulation in grasses and dicots.

In *Medicago* and Arabidopsis, WRKY12 inhibits secondary wall formation though directly binding to the promoter of NST2, while the expression of WRKY12 is auto-repressed (Wang et al., 2010), which is a feature of WRKY signaling (Rushton et al., 2010; Phukan et al., 2016). In grasses, we suggest that WRKY12 may serve as a repressor in a similar way by down-regulating SWNs and auto-regulating itself.

### KNOX, BEL, and OFP Groups of TFs Involved in Secondary Wall Accumulation in GA-Signaling and Organ Development

KNOX and BEL, two subclasses belonging to the TALE (Three Amino acid Loop Extension) homeodomain superclass, are the oldest TF groups diversely represented across the plant kingdom including green and red algae (Mukherjee et al., 2009). Members of class I KNOX genes from Arabidopsis, *Populus*, peach, maize, and switchgrass have been identified to be negative regulators of secondary wall deposition (Mukherjee et al., 2009; Hay and Tsiantis, 2010; Li E. et al., 2012; Townsley et al., 2013; Gong et al., 2014; Liu et al., 2014; Wuddineh et al., 2016). Overexpression of ZmKN1 in maize and tobacco significantly reduced the lignin content and altered lignin composition (Townsley et al., 2013). Partially similarly, a switchgrass PvKN1 (the ortholog of maize ZmKN1) overexpressing line displayed abnormal growth with a slightly reduced lignin content, and altered expression of some structural genes involved in cellulose, hemicellulose, and lignin biosynthesis (Wuddineh et al., 2016). Notably, ChIP-seq and qRT-PCR experiments revealed that ZmKN1 and PvKN1 can reduce the expression of the GA 20-oxidase (*GA20ox*, GA synthesis) gene while inducing the expression of GA 2-oxidase (*GA2ox*, GA catabolism), suggesting the roles of the TFs in modulation of GA signaling and maintenance of GA homeostasis (Bolduc et al., 2012; Wuddineh et al., 2016).

Arabidopsis AtKNAT7, a member of the class II KNOX gene family, and AtBLH6, a member of the BEL gene family, are both also considered as repressors in secondary cell wall biosynthesis (Li E. et al., 2012; Liu et al., 2014). Furthermore, interactions between AtKNAT7/AtBLH6 and AtOFP1 and AtOFP4, two members of the OFP (OVATE FAMILY PROTEIN) family, result in heterodimeric complexes with enhanced activity to repress secondary wall thickening in the interfascicular fibers of inflorescence stems (Li E. et al., 2012; Liu et al., 2014; Liu and Douglas, 2015). As mentioned above, the expression of AtKNAT7 can be induced by the secondary wall-activators AtMYB46/83 and AtMYB61 (Zhong et al., 2008; Romano et al., 2012). This suggests that AtKNAT and the formation of the AtKNAT7-AtBLH6-OFPs multi-protein complex contribute to a negative feedback loop for fine tuning of secondary wall biosynthesis (Li E. et al., 2012; Liu and Douglas, 2015). In rice, overexpressing OsOFP2 causes disruption of vascular bundle arrangement in the stem and lower GA content, through alteration of gene expression associated with lignin biosynthesis, vascular development, and GA synthesis (Schmitz et al., 2015). In addition, yeast two-hybrid assays have proven the interactions between OsOFP2, OsKNAT7 and OsBLH6-like 1 and OsBLH6-like 2 (Schmitz et al., 2015). Considering that OFP is a land plant-specific TF family (Wang et al., 2016), it has been proposed that grasses and dicots have evolved OFP TFs which interact with KNOX and BEL members rooted from the last common ancestors with non-vascular plants, to control vascular development through suppression of GA and lignin biosynthesis (**Figure 1**).

In addition, the BEL-type homeodomain genes contribute to controlling lignin biosynthesis in replum development and seed shattering. AtBLH9 (also named as *REPLUMLESS*, RPL) is a key regulator for determining the orientation of stem growth (Bencivenga et al., 2016). The target genes of AtBLH9 identified by genome-wide ChIP-seq include *AtBGLU45* encoding stem-specific monolignol β-glucosidase (Chapelle et al., 2012) and the S-lignin biosynthesis-specific gene *AtF5H* (Bencivenga et al., 2016). Similarly, the high expression of OsSH5 (the homolog of AtBLH9 in rice) in rice pedicels inhibits the accumulation of lignin content by repressing the expression of lignin biosynthesis genes in the abscission zone (Yoon et al., 2014). This suggests that OsSH5 and AtBLH9 may play similar roles in repressing lignin biosynthesis during organ development.

Though a conserved function of members of the TALE and OFP families may be shared in grasses and dicots for the regulation of secondary wall accumulation, some differences are observed. Overexpression of AtBLH6 in Arabidopsis causes a reduction of secondary wall thickness in interfascicular fibers and a significant repression of stem growth (Liu et al., 2014). Interestingly, overexpressing OsBLH6, the third ortholog of Arabidopsis AtBLH6, in rice causes enhanced secondary wall-development in the stem but similar plant growth compared with the control; an OsBLH6 knock down line exhibits reduced lignin content, especially in the sclerenchyma in stems (Hirano et al., 2013b). The opposite direction of regulation by rice and Arabidopsis BLH6 orthologs in secondary wall accumulation suggests that they have undergone functional specialization after gene duplication.

### C2H2 Group TFs Involved in Secondary Wall Formation

Besides the NAC, MYB and TALE families, the C2H2 family is listed in TF families that have the most abundant members co-expressed with secondary cell wall structural genes in rice and Arabidopsis (Hirano et al., 2013a). One C2H2 member named OsIDD2 was proven to be a negative regulator of secondary wall formation (Huang et al., 2017). Overexpressing OsIDD2 in rice decreases the lignin content with a reduced expression of several structural genes involved in lignin, cellulose, and sucrose biosynthesis. The direct repression of *OsCAD2* and *OsCAD3* expression by OsIDD2 indicates its negative role in lignin accumulation (Huang et al., 2017).

### The E2Fc Group of TFs Coupling Secondary Wall Initiation and Cell Cycle Exit

Secondary cell wall formation is coupled with cell cycle exit because secondary walls are deposited in the cells during the phase when growth stops and differentiation begins (Kwok and Wong, 2003). The inhibition of genes involved in cell division and the activation of genes involved in secondary cell wall biosynthesis occur at the same time in hormone-induced suspension cells of Arabidopsis and switchgrass (Pauwels et al., 2008; Rao et al., 2017). In Arabidopsis, E2Fc, a member of the E2F family, is considered to play a dual regulatory role in cell proliferation and secondary wall formation (del Pozo et al., 2002, 2006; Heckmann et al., 2011). E2Fc and its variants are capable of directly binding to the promoter of the centromere-specific histone in a cell cycle-dependent manner, and to the promoters of several secondary cell wall biosynthesis genes (Taylor-Teeples et al., 2015). E2Fc can activate the expression of VND7 in a dose-dependent manner (Taylor-Teeples et al., 2015), which further triggers a rapid cell death-program and secondary cell wall initiation in the tracheary element-differentiation process (Yamaguchi et al., 2010; Bollhoner et al., 2012). Four *E2F* genes, homologs of known cell cycle regulators in Arabidopsis, show tight coexpression with lignin biosynthesis genes in the time-course of secondary cell wall formation induced by the hormone brassinosteroid in switchgrass suspension cells (Rao et al., 2017). Their role in secondary cell wall formation and cell proliferation is worth exploring in the future.

### Lineage-Specific TFs

Besides the examples mentioned above, additional functional divergences in cell wall regulation have been reported in monocots and dicots. For instance, homologous overexpression of the *SHN* gene in Arabidopsis (Aharoni et al., 2004; Shi et al., 2011), rice (Wang et al., 2012; Zhou et al., 2014), and switchgrass (Wuddineh et al., 2015) provides evidence for its function in wax biosynthesis. However, the heterologous expression of *AtSHN* in rice resulted in the downregulation of lignin biosynthesis genes and upregulation of cellulose synthesis genes, which led to a significant increase in cellulose and decrease in lignin content (Ambavaram et al., 2011). The discrepancy between homologous and heterologous expression of SHN may reflect the divergence between regulatory mechanisms of cell wall development in monocots and dicots.

Though sharing many TFs rooted from the last common ancestor, monocots and dicots have developed some lineage-specific TFs through gene duplication. For instance, AtMYB75 belongs to a dicot-specific group with orthologs found in poplar, but not in grasses (Zhao and Bartley, 2014). AtMYB75 is a master switch to control the shift from secondary wall formation to anthocyanin accumulation via repression of secondary wall-related cellulose synthase genes and lignin biosynthesis genes and activation of the late anthocyanin biosynthetic genes in a light-dependent manner (Bhargava et al., 2010; Li et al., 2016). Monocots may have evolved other TFs participating in light-controlled secondary wall accumulation. Zhao and Bartley (2014) have identified several grass-specific TF clades. Candidates in these clades may have potential roles in secondary wall regulation in a grass-specific manner.

Recent tissue-specific and time-course transcriptome analyses from sorghum, *Miscanthus lutarioriparius*, and switchgrass have revealed 100s of TF genes whose expression is highly correlated with the dynamic process of lignification (Hu et al., 2017; Kebrom et al., 2017; Rao et al., 2017; Yan et al., 2017), providing more TF candidates in grasses for future functional identification.

## Conclusion

According to current research, grasses and dicots share a conserved transcriptional regulatory network for secondary wall biosynthesis, nevertheless with many grass-specific features. The differences and commodities in the transcriptional networks for secondary cell wall regulation between Arabidopsis and grasses are summarized in **Figure 1** and **Table 1**, and details of the individual TFs are listed in **Supplementary Table S1**. The differences may be caused by changes in spatial expression of TFs, *cis*-regulatory element composition of structural genes and sub-functionalization after gene duplication. Considering the economic and ecological importance of the grass family, further research is needed to better understand the grass-specific transcriptional regulation of secondary cell wall development.

**Table 1:** Summary of the commonalities and differences in transcriptional regulation of secondary wall formation in Arabidopsis and grasses.

TF group	Arabidopsis	Grasses	Reference
SWN	Binds to the SNBE motif; activates MYB46/83 and lower-level MYBs; induces cellulose, xylan, and lignin		Zhao et al., 2010; Zhong et al., 2010a,b, 2011, 2015; Wang et al., 2011; Valdivia et al., 2013; Yoshida et al., 2013; Xiao et al., 2017
	Some SWNs are expressed only in vessels or fibers	Expressed in both vessels and fibers	Zhong et al., 2010a, 2011, 2015; Valdivia et al., 2013; Yoshida et al., 2013; Xiao et al., 2017
	SND1 shows auto-activation	Rice OsSWN2 variant may negatively regulate OsSWN1 and OsSWN2	Wang et al., 2011; Yoshida et al., 2013

MYB46/83	Binds to the SMRE motif; activates lower-level MYBs; induces cellulose, xylan, and lignin		Zhong et al., 2007a, 2011, 2015; McCarthy et al., 2009; Ko et al., 2012; Kim et al., 2013

MYB58/63	Specifically induces lignin	Regulates both lignin and secondary wall-related cellulose in rice and sorghum	Zhou et al., 2009; Zhao and Dixon, 2011; Noda et al., 2015; Scully et al., 2016

MYB55/61	Actives genes involved in lignin biosynthesis (CCoAOMT7) and pectin modification (PME)	Actives lignin and cellulose biosynthesis in rice	Newman et al., 2004; Romano et al., 2012; Hirano et al., 2013b; Huang et al., 2015

MYB103	Alters lignin composition via regulation of AtF5H	No evidence to show specific regulation of F5H; increases total lignin and cellulose content in rice	Hirano et al., 2013b; Ohman et al., 2013; Yang et al., 2014; Ye et al., 2015

MYB4/32	MYB4 represses the lignin biosynthesis genes C4H and 4CL	Share common target of COMT but display lineage-specific suppression of other lignin biosynthesis genes in grasses	Jin et al., 2000; Sonbol et al., 2009; Fornalé et al., 2010; Shen et al., 2012; Vélez-Bermúdez et al., 2015; Agarwal et al., 2016

WRKY12	Represses lignification		Wang et al., 2010; Gallego-Giraldo et al., 2016; Yang et al., 2016

KNOX	Negatively regulates secondary wall deposition		Mukherjee et al., 2009; Hay and Tsiantis, 2010; Li E. et al., 2012; Townsley et al., 2013; Gong et al., 2014; Liu et al., 2014; Wuddineh et al., 2016

KNAT/BEL/OFP	May form KNAT-BLH-OFP multi-protein complex as a negative regulator of secondary wall development		Li E. et al., 2012; Liu et al., 2014; Liu and Douglas, 2015; Schmitz et al., 2015

BLH9	Represses lignin accumulation		Chapelle et al., 2012; Yoon et al., 2014; Bencivenga et al., 2016

BLH6	Represses secondary wall development	Induces secondary wall development in rice	Hirano et al., 2013b; Liu et al., 2014

C2H2	NA^∗^	OsIDD2 represses secondary wall development in rice	Huang et al., 2017

E2Fc	Triggers cell proliferation and secondary wall formation	NA^∗^	del Pozo et al., 2002, 2006; Heckmann et al., 2011; Taylor-Teeples et al., 2015

*NA^∗^, members of the C2H2 family in Arabidopsis and E2Fc in grasses show co-expression with secondary wall structural genes but have not been proven to be regulators of secondary wall formation (Hirano et al., 2013a; Rao et al., 2017).*

## Author Contributions

XR collected data from literature and wrote the manuscript. RD revised the article.

## Conflict of Interest Statement

The authors declare that the research was conducted in the absence of any commercial or financial relationships that could be construed as a potential conflict of interest.

## Supplementary Material

FIGURE S1 Phylogenetic analysis of SWNs from Arabidopsis and five grass species.

FIGURE S2 Phylogenetic analysis of secondary wall-related MYBs from Arabidopsis and five grass species.

TABLE S1 Transcription factors involved in grass secondary cell wall formation.
