# Supplementary figures and images for: Current Models for Transcriptional Regulation of Secondary Cell Wall Biosynthesis in Grasses
Source: Front Plant Sci. 2018 Apr 4;9:399. doi: 10.3389/fpls.2018.00399 (PMC5893761; doi:10.3389/fpls.2018.00399)

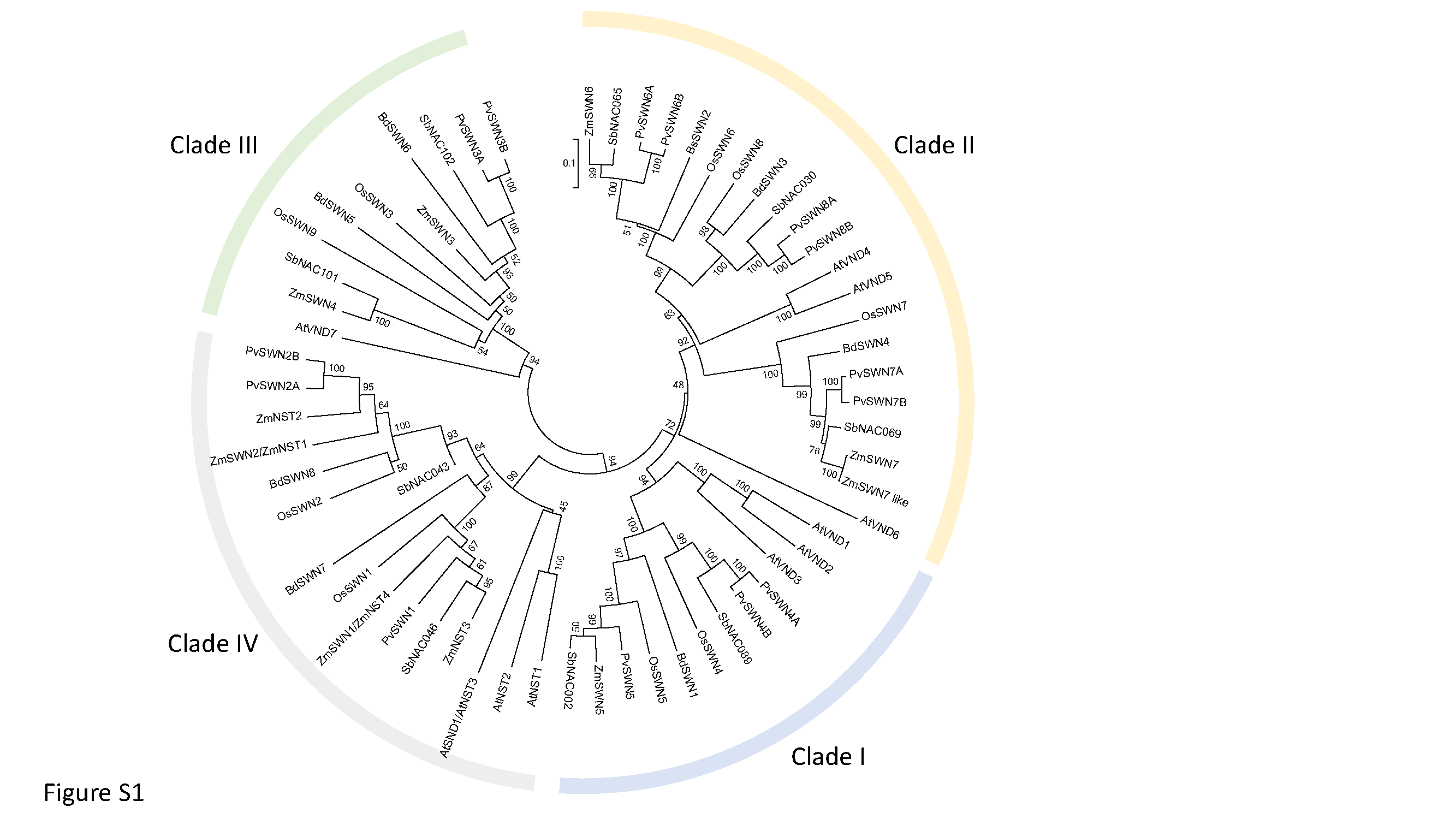

Supplement: FIGURE S1 — Phylogenetic analysis of SWNs from Arabidopsis and five grass species. [file Image_1.TIFF]

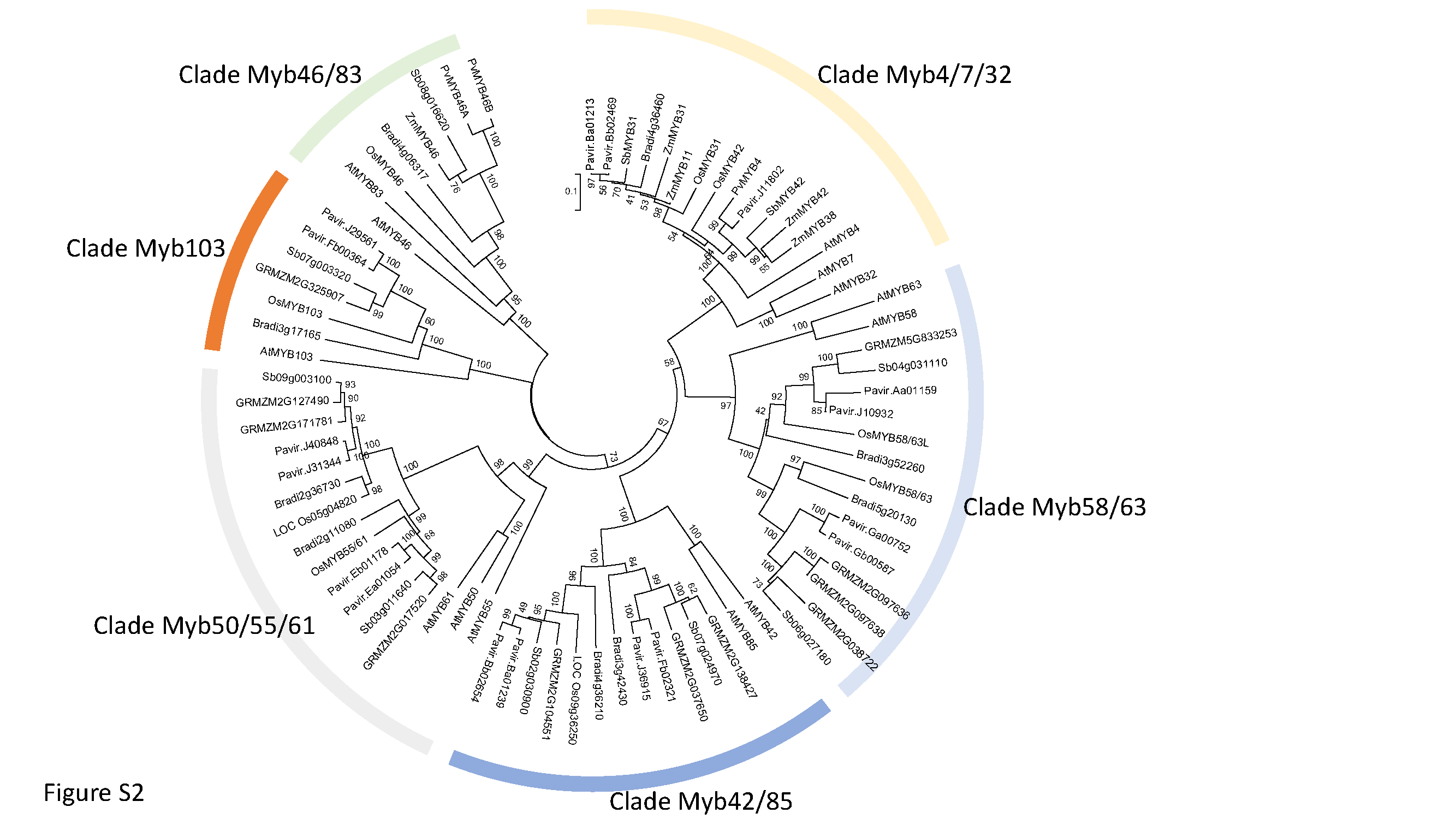

Supplement: FIGURE S2 — Phylogenetic analysis of secondary wall-related MYBs from Arabidopsis and five grass species. [file Image_2.TIFF]
