# Supplementary material for: Current Models for Transcriptional Regulation of Secondary Cell Wall Biosynthesis in Grasses
Source: Front Plant Sci. 2018 Apr 4;9:399. doi: 10.3389/fpls.2018.00399 (PMC5893761; doi:10.3389/fpls.2018.00399)
Supplement: TABLE S1 — Transcription factors involved in grass secondary cell wall formation. [file Table_1.DOCX]

| TF family | Gene name | Transcript ID | Regulator | References |
| --- | --- | --- | --- | --- |
| ***Zea mays*** | | | | |
| WRKY | ZmWRKY12 | GRMZM2G123387 | repressor | Gallego-Giraldo et al., 2015 |
| NAC | ZmSWN1/ZmNST4 | GRMZM2G171395 | activator | Zhong et al., 2011; Zhu et al., 2012; Xiao et al., 2017 |
|  | ZmNST3 | GRMZM2G091490 | activator |  |
|  | ZmNST2 | GRMZM2G092465 | activator |  |
|  | ZmSWN2/ZmNST1 | GRMZM2G069047 | activator |  |
|  | ZmSWN3 | GRMZM2G052239 | activator |  |
|  | ZmSWN4 | AC212859.3_FG008 | activator |  |
|  | ZmSWN5 | GRMZM2G025642 | activator |  |
|  | ZmSWN6 | GRMZM2G178998 | activator |  |
|  | ZmSWN7 | GRMZM2G041668 | activator |  |
|  | ZmSWN7_like | GRMZM2G440219 | activator |  |
| MYB | ZmMYB46 | GRMZM2G052606 | activator | Zhong et al., 2011 |
|  | ZmMYB31 | GRMZM2G050305 | repressor | Fornale et al., 2006; Sonbol et al., 2009; Fornale et al., 2010; Vélez-Bermúdez et al., 2015; Agarwal et al., 2016 |
|  | ZmMYB11 | GRMZM2G000818 | repressor |  |
|  | ZmMYB42 | GRMZM2G419239 | repressor |  |
|  | ZmMYB38 | GRMZM2G084583 | repressor |  |
| TIFY | ZmZML2 | GRMZM2G058479 | repressor | Vélez-Bermúdez et al., 2015 |
| TALE | ZmKN1 | GRMZM2G017087 | repressor | Townsley et al., 2013 |
| ***Sorghum bicolor*** | | | | |
| NAC | SbNAC002 | Sb01g048730 | activator | Zhu et al., 2012 |
|  | SbNAC030 | Sb04g033570 | activator |  |
|  | SbNAC043 | Sb07g001550 | activator |  |
|  | SbNAC046 | Sb10g002120 | activator |  |
|  | SbNAC065 | Sb06g023780 | activator |  |
|  | SbNAC069 | Sb10g000460 | activator |  |
|  | SbNAC089 | Sb01g030760 | activator |  |
|  | SbNAC101 | Sb06g034010 | activator |  |
|  | SbNAC102 | Sb07g000470 | activator |  |
| MYB | SbMYB60 (ortholog of AtMYB58/63) | Sb04g031110 (Sobic.004G273800) | activator | Scully et al., 2016 |
|  | SbMYB42 | Sb07g024890 | repressor | Agarwal et al., 2016 |
|  | SbMYB31 | Sb02g031190 | repressor |  |
| ***Oryza sativa*** | | | | |
| NAC | OsSWN1 | LOC_Os06g04090 | activator | Zhong et al., 2011; Hirano et al., 2013; Yoshida et al., 2013; Huang et al., 2015 |
|  | OsSWN2 (NAC29) | LOC_Os08g02300 | activator |  |
|  | OsSWN3 (NAC31) | LOC_Os08g01330 | activator |  |
|  | OsSWN4 | LOC_Os10g38834 | activator |  |
|  | OsSWN5 | LOC_Os03g03540 | activator |  |
|  | OsSWN6 | LOC_Os04g45340 | activator |  |
|  | OsSWN7 | LOC_Os06g01480 | activator |  |
|  | OsSWN8 | LOC_Os02g42970 | activator |  |
|  | OsSWN9 | LOC_Os04g59470 | activator |  |
| MYB | OsMYB46 | LOC_Os12g33070 | activator | Zhong et al.,2011 |
|  | OsMYB31 | LOC_Os09g36730 | repressor | Agarwal et al., 2016 |
|  | OsMYB42 | LOC_Os08g43550 | repressor |  |
|  | OsMYB42/85 | LOC_Os09g36250 | activator | Hirano et al., 2013; Noda et al., 2015; Huang et al., 2015 |
|  | OsMYB58/63 | LOC_Os04g50770 | activator |  |
|  | OsMYB58/63-L | LOC_Os02g46780 | activator |  |
|  | OsMYB55/61 | LOC_Os01g18240 | activator |  |
|  | OsMYB103 | LOC_Os08g05520 | activator | Yang et al., 2014; Ye et al., 2015 |
| TALE | OsSH5 | LOC_Os05g38120 | repressor | Yoon et al., 2014 |
|  | OsBLH6 | LOC_Os03g06930 | activator | Hirano et al., 2013 |
|  | OsBLH6-like 1 | LOC_Os03g47740 |  | Schmitza et al., 2015 |
|  | OsBLH6-like 2 | LOC_Os12g43950 |  |  |
|  | OsKNAT7 | LOC_Os03g03164 | repressor |  |
| OFP | OsOFP2 | LOC_Os01g43610 | repressor |  |
| C2H2 | OsIDD2 | LOC_Os01g09850 | repressor | Huang et al., 2017 |
| ***Panicum virgatum*** | | | | |
| WRKY | PvWRKY12 | Pavir.Ga00648 | repressor | Gallego-Giraldo et al., 2015 |
| NAC | PvSWN1 | Pavir.J07835 | activator | Zhong et al., 2015 |
|  | PvSWN2A | Pavir.J20698 | activator |  |
|  | PvSWN2B | Pavir.J21162 | activator |  |
|  | PvSWN3A | Pavir.J20890 | activator |  |
|  | PvSWN3B | Pavir.J31179 | activator |  |
|  | PvSWN4A | Pavir.Ib02477 | activator |  |
|  | PvSWN4B | Pavir.Ia02924 | activator |  |
|  | PvSWN5 | Pavir.Ib00161 | activator |  |
|  | PvSWN6A | Pavir.J07126 | activator |  |
|  | PvSWN6B | Pavir.Gb00744 | activator |  |
|  | PvSWN7A | Pavir.J26987 | activator |  |
|  | PvSWN7B | Pavir.Da02426 | activator |  |
|  | PvSWN8A | Pavir.J09314 | activator |  |
|  | PvSWN8B | Pavir.J39804 | activator |  |
| MYB | PvMYB4 | Pavir.J16675 | repressor | Shen et al., 2011; Shen et al., 2013 |
|  | PvMYB46A | Pavir.J11191 | activator | Zhong et al., 2015 |
|  | PvMYB46B | Pavir.Ca02370 | activator |  |
| TALE | PvKN1a | Pavir.Ca01130 | repressor | Wuddineh et al., 2016 |
|  | PvKN1b | Pavir.J13005 | repressor |  |
| ***Brachypodium distachyon*** | | | | |
| NAC | BdSWN1 | Bradi1g76732 | activator | Valdivia et al., 2013 |
|  | BdSWN2 | Bradi5g16917 | activator |  |
|  | BdSWN3 | Bradi3g50067 | activator |  |
|  | BdSWN4 | Bradi1g52187 | activator |  |
|  | BdSWN5 | Bradi5g27467 | activator |  |
|  | BdSWN6 | Bradi3g13117 | activator |  |
|  | BdSWN7 | Bradi1g50057 | activator |  |
|  | BdSWN8 | Bradi3g13727 | activator |  |
